# The economic value of mussel farming for uncertain nutrient removal in the Baltic Sea

**DOI:** 10.1371/journal.pone.0218023

**Published:** 2019-06-14

**Authors:** Ing-Marie Gren

**Affiliations:** Department of Economics, Swedish University of Agricultural Sciences, Uppsala, Sweden; Helmholtz-Zentrum fur Ozeanforschung Kiel, GERMANY

## Abstract

Mussel farming has been recognised as a low cost option for mitigating damage caused by eutrophication in the Baltic Sea. However, uncertain nutrient removal owing to weather and environmental conditions at the mussel farm site has not been previously considered. The purpose of this study was to estimate whether mussel farming has cost advantages even in conditions of uncertainty. To this end, the replacement cost method was used for the valuation of ecosystem services and a numerical cost minimisation model was constructed based on the safety-first approach to account for uncertainty in nutrient removal. This study showed that the value of mussel farming depends on the cost at the farm, and the impact on the mean and variability of nutrient removal in relation to other abatement measures. The study also pointed out the need of data on the decision makers’ risk attitudes and measurement of uncertainty. The application to the Baltic Sea showed that the total value of mussel farming increased from 0.34 billion Euro/year to 0.41 or 1.21 billion Euro when accounting for uncertainty depending on assumption of probability distribution. The increase was unevenly distributed between the Baltic Sea countries, with it found to be lower for countries equipped with highly productive mussel farms and long coastlines.

## Introduction

Like many other seas and lakes, the Baltic Sea suffers from eutrophication due to excess loads of nitrogen and phosphorous. Damage from eutrophication is manifested as increased frequency in the blooming of toxic algae and changes in the composition in fish species, usually to the detriment of commercial species (*e*.*g*. [1]). The Baltic Sea has the world’s largest sea bottoms with no biological life [2]. Awareness of this damage was raised in early 1970s, but progress towards making improvements is slow. One reason for this is the difficulty of regulating nutrients from the agricultural sector, which accounts for 60% of the nitrogen loads and 50% of the phosphorus loads [3]. Mussel farming has been suggested as a promising abatement option since it reduces the nutrient content in the sea through the cultivation and harvesting of mussels, which can be used as feed, food or an energy source [4,5,6].

One disadvantage of mussel farming, however, is the uncertainty associated with how the technology functions, for example storms might damage the platforms or the growth of larvae attached to the ropes is uncertain because of temperatures and currents at the site. It may even create negative nutrient removal because farmed mussels excrete nutrients at the site, which can create a leakage of ammonia and phosphorus from the sediments [7]. Despite this well-known disadvantage of mussel farms, no study has been undertaken on the cost-effectiveness of mussel farming as a nutrient-cleaning option taking this uncertainty into consideration.

The purpose of this study was to assess the economic value of mussel farming with uncertain nutrient removal in the Baltic Sea. To this end, two different methods were combined: the replacement cost method to estimate its value, and the safety-first approach to consider uncertainty. The replacement cost method in valuing ecosystem services implies that the service, in this case nutrient cleaning by mussel farming, is valued according to the contribution it makes to cost savings in reaching predetermined environmental targets (*e*.*g*. [5]). The safety-first approach is most often used when decision-makers are concerned with reaching predetermined targets, which in this study were nutrient load targets. This concern is expressed in terms of the probabilities of reaching the target [8]. In addition to nutrient load targets, the decision-maker thus has to choose a minimum acceptable probability for reaching the target [9, 10, 11]. By applying chance-constrained programming, the probabilistic target is expressed in deterministic terms, which enables numerical optimisation.

A few studies have evaluated the economic feasibility of mussel farming [4,5,6,12,13,14,15]. Four of them, Lindahl *et al*. [4], Schernewski *et al*. [6], Ngyen *et al*. [12] and Schernewski *et al*. [14] performed cost-benefit analyses by comparing the cost and benefits (from sales of mussels) and calculated minimum payments for nutrient cleaning necessary for a mussel farm to make no net loss. Peterson *et al*. [13] compared costs of nutrient removal by mussel farms with other abatement measures. Gren *et al*. [5,15] estimated the value of mussel farming by applying the replacement cost method, but did not consider uncertainty in abatement. Application of the replacement cost method requires a cost-effectiveness analysis since the value of any new technology is calculated as the difference in minimum cost for achieving certain nutrient targets. There is a relatively large body of literature on cost-effective nutrient abatement of nutrient loads to the Baltic Sea (see the review in Elofsson [16], but only a few consider uncertainty in the abatement of any technology [9, 10]. Both these studies applied the safety-first approach in the same way as in the present study where costs are minimized for reaching probabilistic nutrient load targets.

The safety-first approach has a long tradition in economics where it has been applied to cost effective provision of food resources (*e*.*g*. [8]), agriculture and forest measures (*e*.*g*. [17, 18]), climate change mitigation (*e*.*g*. [19, 20]), and water quality management (*e*.*g*. [9,10,11]). Similar to the present study, a common difficulty for these studies has been to find data on decision makers’ attitudes towards the risk of not attaining targets and measurement of uncertainty in the effects of the measures. Following the approaches applied in these studies, the impact of the lack of data on the estimated value of mussel farming is considered by allowing for different measurements of uncertainty and risk aversions.

In the author’s view, the main contribution of the present study to the literature is the valuation of mussel farming in the presence of uncertain abatement by combining the replacement cost method for valuing ecosystem services with the safety-first approach to account for uncertainty. Another contribution is the consideration of different measurements of uncertainty. The study is organised as follows. First, there is a presentation of the conceptual approach used to identify conditions for a positive economic contribution by mussel farming as a nutrient-cleaning device. This is then followed by a description of the data used in the chance-constrained model for cost-minimisation. The results are presented in Section 4, and then discussed in Section 5, before the study ends with conclusions.

## Conceptual approach

According to the replacement cost method, a cleaning technology has a value only if its introduction reduces the total abatement cost for achieving a certain nutrient load reduction target (*e*.*g*. [5]). The value is then calculated as the difference in total minimum costs for reaching the targets with and without mussel farming as a nutrient-removal option. Since this value is determined by the construction of the cost minimisation model under uncertain nutrient abatement, it is briefly presented below.

The catchment of the Baltic Sea includes *i = 1*,..,*g* countries, each of which has access to measures to abate nutrient *U*, where *N = nitrogen* and *P = phosphorus*, by mussel farming, *A*^*i*,*U*,*M*^ and other abatement measures, *A*^*i*,*U*,*O*^. Both these nutrient-reduction options are uncertain, which is described as:

$$A^{i,U,M}=c^{i,U,M}A^{i,M}+\varepsilon^{i,U,M}$$ (1)

$$A^{i,U,O}=c^{i,U,O}A^{i,O}+\varepsilon^{i,U,O}$$ (2)

where *c*^*i*,*U*,*M*^ and *c*^*i*,*U*,*O*^ are the conversion parameters of mussel biomass and other abatement measures into nutrients, and *ε*^*i*,*U*,*M*^ and *ε*^*i*,*U*,*O*^ are the additive uncertainty terms in nutrient reductions. Total reduction in the load of a nutrient *U* to the Baltic Sea is then written as:

$$A^{U}=\sum_{i}\;A^{i,U,M}+A^{i,U,O}$$ (3)

Nutrient reduction targets for the Baltic Sea are set for each nutrient in international agreements, ${\bar{A}}^{U}$, by the Baltic Sea Action Plan (BSAP) in HELCOM [21]. In the safety-first decision framework used in this study a decision-maker also has to decide on the minimum probability, *α*^*U*^, at which the target should be achieved. This has not been made by HELCOM in any of its intergovernmental agreements. The probabilistic reduction target is written as:

$$prob(A^{U}\geq {\bar{A}}^{U})\geq \alpha$$ (4)

Chance-constrained programming is used to solve the cost-minimisation problem with a probabilistic constraint (*e*.*g*. [22]). This means that Eq (4) is transformed into a deterministic equivalent as (*e*.*g*, [22]):

$$E[A^{U}]-\phi^{\alpha U}Var{\left(A^{U}\right)}^{1/2}\geq {\bar{A}}^{U}$$ (5)

where *E[A*^*U*^*]* is the mean abatement and *Var(A*^*U*^*)* is the variance. Eq (5) shows that the nutrient target restriction becomes tighter because of the risk discount shown by the second term on the left-hand side of the inequality sign in the equation. This means that more abatement is needed in order to ensure achievement of the targets, which raises the total abatement costs. This cost of uncertainty is determined by the level of *ϕ*^*αU*^ and *Var(A*^*U*^*)*.

The parameter *ϕ*^*αU*^ reflects the decision-maker’s risk aversion against non-attainments of the abatement targets, when *ϕ*^*αU*^>0 the decision maker is concerned about reaching the targets and *ϕ*^*αU*^ = 0 otherwise. The level of *ϕ*^*αU*^ is determined by the choice of probability of reaching the targets, *α*^*U*^, and the probability distribution. A common approach is to assume a normal probability, and *ϕ*^*αU*^ is then determined where $\int_{-\infty }^{\phi^{\alpha ,U}}f(\phi^{\alpha ,U})d\phi^{\alpha ,U}=\alpha^{U}$, the calculations of which can be found in students’ t-tables where, for example, *ϕ*^*αU*^ = 1.26 when *α*^*U*^ *= 0*.*9* (see *e*.*g*. Taha [22]). However, due to lack of empirical evidences there are no a priori expectations about the probability distributions. This is an argument in favour of a more flexible distribution, Chebyshev’s inequality without assumptions on the shape of the probability distribution, and *ϕ*^*αU*^ is then determined where *ϕ*^*αU*^ = 1/(1−*α*^*U*^)^1/2^ (see McCarl [23] for more details). The level of *ϕ*^*αU*^ for *α*^*U*^ *= 0*.*9* is then 3.16, which is considerably larger than for the same probability choice with a normal distribution. This, in turn, means that the costs of uncertainty is higher than for a normal distribution. Calculations are made for both probability distributions in Section 4.

With respect to *Var(A*^*U*^*)*, it is allowed for dependency between *A*^*i*,*U*,*M*^ and *A*^*i*,*U*,*O*^ in this study. Because of the focus on the role of mussel farming for costs of nutrient abatement under uncertainty, simplifications are made by assuming that uncertainty in abatement is attached to each country. The variance is then written as:

$$Var(A^{U})=\sum_{i}\;Var(A^{i,U,M})+Var(A^{i,U,O})+Cov(A^{i,U,M},A^{i,U,O})$$ (6)

The variance in abatement is thus the sum of the own variances in abatement by mussel farming and other abatement measures plus the covariance. The covariance shows if an how abatement by the two classes of abatement measure varies in a systematic way such that abatement is high or low at the same time (a positive covariance), or high for one and low for another (a negative covariance). The covariance is zero in the absence of any such covariation. There can be a negative covariation if the content of nutrient in the waters at a mussel farming site depends on the load of nutrient from the catchment, which can be high when abatement is low. If so, mussel farming can be regarded as a hedge against large nutrient loads from land, which has been demonstrated for construction of wetlands (Byström *et al*. [9]). On the other hand, when the covariation is positive mussel farming reinforces relatively high loads from land by showing a low abatement. There exists no empirical evidence for any of these covariations, and calculations are therefore made for all three possibilities in Section 4.

Each class of measure gives rise to abatement costs, *C*^*i*,*M*^ = *C*^*i*,*M*^*(A*^*i*,*M*^*)* and *C*^*i*,*O*^ = *C*^*i*,*O*^*(A*^*i*,*O*^*)*, which are specific to each country and increasing and convex in their arguments, *i*.*e*. costs are increasing at a non-decreasing rate. However, mussel farming and all other abatement measures face capacity constraints. For example, mussel farming cannot be placed everywhere along the coast, and there are restrictions on the amount of abatement in agriculture and at sewage treatment plants. This is of particular relevance for the static model used in this study, which did not consider technological development. Further, large reductions in nutrient loads may cause dispersal effects into the rest of the economies by affecting prices of *e*.*g*. agricultural input and outputs, which are not captured by the model in this study. Such considerations would require partial or general equilibrium model, which are much used to evaluate agricultural policies (*e*.*g*. [24]). The capacity constraint for each class of measure is then written as:

$$A^{i,M}\leq {\bar{A}}^{i,M};\;A^{i,O}\leq {\bar{A}}^{i,O}$$ (7)

A Baltic Sea perspective is applied where the decision problem is formulated as minimising the costs for all countries of reaching the nutrient abatement targets according to:

$$\begin{array}{l} Min\;C=\sum_{i}\;C^{i,,M}+C^{i,,O}\;subject\;to\;equations\;(4),(6)\;and\;(7)\\ A^{i,M},A^{i,O} \end{array}$$ (8)

The value of mussel farming in combatting eutrophication is determined by the first-order conditions for a cost-effective choice of mussel farming and other abatement measures. As shown in S1 Appendix, mussel farming has a value for reaching a target on one nutrient *U* only when:

$$\frac{MC^{i,M}}{c^{i,U,M}-MR^{i,U,M}}<\frac{MC^{i,O}}{c^{i,U,O}-MR^{i,U,O}}\;for\;any\;A^{i,M}>0$$ (9)

where *MC*^*i*,*M*^ and *MC*^*i*,*O*^ are the marginal cost of mussel production and abatement by other measures respectively, and *MR*^*i*,*M*^ and *MR*^*i*,*O*^ are the marginal impacts on the risk discount in Eq (6). Without any marginal impacts on risk, i.e. when *MR*^*i*,*M*^ = *MR*^*i*,*O*^ *= 0*, mussel farming is included in a cost-effective solution when the marginal abatement cost of a nutrient *U* by mussel farming, i.e. $\frac{MC^{i,M}}{c^{i,U,M}}$ evaluated at zero mussel farming, is lower than the marginal abatement cost of the same nutrient by other abatement measures, i.e. $\frac{MC^{i,O}}{c^{i,U,O}}$, for any positive level of *A*^*i*,*M*^. For example, assume that the constant marginal cost of phosphorus removal by mussel farming amounts to Euro 300/kg P. Mussel farming is then a cost-effective option only if the upper limit of the marginal abatement cost of other measures is above Euro 300/kg P abatement.

The consideration of uncertainty, *i*.*e*. when *MR*^*i*,*U*,*M*^*>0* and *MR*^*i*,*U*,*O*^*>0*, increases the marginal cost of nutrient reduction for mussel farming and other abatement measures because of the need to abate more compared with no uncertainty. This then means that inclusion of uncertainty increases (decreases) the value of mussel farming when *MR*^*i*,*U*,*M*^*<MR*^*i*,*U*,*O*^ (*MR*^*i*,*U*,*M*^*>MR*^*i*,*U*,*O*^*)*. In other words, the value of mussel farming increases when the marginal impact on the risk of mussel farming is lower than that for other abatement measures and *vice versa*.

However, as shown in Eqs (1)–(3), both mussel farming and other abatement measures reduce both nutrients. The principle analytical conclusion presented in Eq (9), remains the same but the first-order conditions become more involved (S1 Appendix). An additional factor to consider is then the multifunctional abatement of both nutrient by mussel farming in relation to other abatement measures. Mussel farming then has a cost advantage if its simultaneous impacts on both nutrients, including impacts on uncertainty, is relatively higher than that for other abatement measures. The theoretical analysis thus points out the need to assess abatement costs, mean and variability in abatement of mussel farming and other abatements measures in order to estimate the value of mussel farming as a cleaning device.

## Description of data in the cost minimisation model

The conceptual approach presented in Section 2 shows that data are needed on nutrient loads in the business-as-usual (BAU) scenario, nutrient removal and costs of mussel farming and other abatement measures and their capacity constraints, nutrient reduction targets and achievement probabilities. The most up-to-date data on BAU loads of nutrients from the various countries with coastal zones on the Baltic Sea are from HELCOM [25], which reports nutrient loads for 2010. The total load of nitrogen is 895 ktonnes N and that of phosphorus is 35.8 ktonnes P (Table 1). Poland accounts for the largest amount of both these nutrients with 33% of total N load and 40% of total P load. It was assumed that the abatement capacity in each country corresponded to 70% of the BAU loads. Measurements of uncertainty in decreases in these loads were obtained from Elofsson [10], who calculated the coefficient of variation (*i*.*e*. standard deviation divided by the mean load) for loads from the different catchments in the Baltic Sea.

**Table 1:** Average loads of nitrogen (N) and phosphorus (P), coefficient of variation (CV) in N and P loads, potential N and P removal by mussel farming, and CV in nutrient removal by mussel farming for the countries around the Baltic Sea.

	Nutrient load ktonne^a^ N P		CV in nutrient loads^b^ N P		Maximum removal by mussels, ktonne^c^ N P		CV in nutrient removal by mussel farms^d^
DEN	57	1.80	0.21	0.27	5.51	0.44	0.25^e^
EST	29	0.67	0.18	0.18	0.60	0.05	0.14^f^
FIN	72	2.97	0.22	0.23	0	0	0
GER	63	0.60	0.20	0.20	5.08	0.41	0.25^e^, 0.21^g^
LAT	85	3.11	0.20	0.21	0.55	0.05	0.21^g^
LIT	61	2.33	0.16	0.15	0.10	0.01	0.21^g^
POL	301	14.49	0.25	0.22	0.70	0.06	0.21^g^
RUS	108	6.21	0.26	0.40	0.16	0.02	0.21^g^
SWE	119	3.65	0.25	0.23	3.35	0.27	0.25^e^, 0.14^f^
Total	895	35.83			15.35	1.31	

^a^HELCOM [25]

^b^Elofsson [7]

^c^1% and 0.08% content of N and P respectively in Kattegat/Danish Straits [31] and 0.75% and 0.06% content of N and P respectively in Baltic Proper [32] of the biomass production displayed in S2 Table

^d^assumed to be the same for nitrogen and phosphorus and calculated by assuming a normal distribution and that the range of biomass includes 95% of all observations [5]

^e^Kattegat and the Danish Straits

^f^North Baltic Proper

^g^South Baltic Proper

DEN (Denmark), EST (Estonia), FIN (Finland, GER (Germany), LAT (Latvia), LIT (Lithuania), POL (Poland), RUS (Russia), SWE (Sweden)

Data on nutrient removal by mussel farms on the large scale of the Baltic Sea are not readily available. Among other things, mussel growths depends on the salinity level, which differs along the coastal zones in the Baltic Sea (*e*.*g*. [7]). In the present study it was assumed that mussels cannot grow and provide a nutrient abatement option in the northern marine basins of the Baltic Sea (Bothnian Bay, Bothnian Sea, Gulf of Finland in S1 Fig) due to unfavourable weather conditions and salinity levels below 6 practical salinity units (*e*.*g*. [26]). The salinity levels are highest in the Kattegat and Danish Straits, and differ in the northern and southern part of the largest basin, Baltic Proper. The biomass production per mussel farm therefore differs in these basins. There is a large body of studies measuring mussel growth and nutrient removal under different environmental conditions including salinity (*e*.*g*. [27,28]), but they are not related to the size of a mussel farm as measured in sea cover area, which is needed for this study.

Production of mussel at a mussel farm of a certain size depends not only on environmental conditions but also on production technology, which includes bottom culture, ropes, and smart farm systems. Calculations have been made for the common longline technology [4,5,13,14,28,29] and for a new technology, the smart farm system, which allows for farming in open waters and sheltered areas [12]. Most of the calculations are made for mussel farms located at the east coast of Denmark and at the west coast of Sweden, and the production varies between 60 and 594 tonnes of mussel/ha and year (S1 Table). The studies on Baltic Proper present calculations ranging between 40 and 167 tonnes of mussels/ha and year.

To the best of the author’s knowledge, there has only been one study examining the production potential of mussel farms in the coastal zones of all these basins [5], data from which was used in the present study (S2 Table). The average production in Kattegat and the Sound basin was 350 tonnes/ha and year which is close to the average of results reported in other studies. The Baltic Proper was divided into North and South Baltic Proper because of the differences in salinity levels, and the production in the South Baltic Proper was assumed to be 160 tonnes/ha and year which is in the upper range of other studies’ estimates. There were no published calculations of similar production in the North Baltic Proper, and it was therefore assumed that it amounts to 140 tonnes/ha and year.

The potential total production depends on production per mussel farm and areas suitable for mussel farming. The long-line technology requires water depths between 6 and 25 m and a maximum average current speed of 5 cm/s [4]. In addition, there might be restrictions imposed by protected marine areas and for recreational purposes (*e*.*g*. [14]). These restrictions can be less severe for offshore mussel farming, which can be located almost 20 km from the coast (*e*.*g*. [30]). There has been no study on the area of potential sites of mussel farms in the Baltic Sea, and therefore the simplifying assumption was made that they can be placed in a maximum area corresponding to 0.5% within 1 nm (1.85 km) of the coasts along the countries’ straight coastlines. The main possible mussel production (71%) takes place in the coastal zones of Kattegat and the Danish Straits owing to the higher productivity per farm and relatively long straight coast lines (S2 Table).

In marine waters the removal of nitrogen and phosphorus are 1% and 0.08% of the biomass respectively (*e*.*g*. [31]). The corresponding removal in the Baltic Proper is regarded as lower because of the lower salinity level, and amounts to 0.75% for nitrogen and 0.06% for phosphorus [32]. When multiplying these nutrient contents with the biomass production in S2 Table, the maximum nitrogen removal amounts to 15.35 ktonnes and phosphorus removal to 1.31 ktonnes (Table 1). There are no data on the coefficient of variation in nutrient removal by mussel farm, and it was therefore assumed that they are the same for nitrogen and phosphorus, and were calculated assuming that 95% of removal outcomes are found within the range of mussel biomass production per farm (Table 1).

There exist no data on the covariation between nutrient removal by mussel farming and other abatement measures. In this study, the covariation is measured as correlation coefficients which are assumed to be the same for all countries. Calculations are made for assumptions of three scenarios; correlation coefficient of 0, -0.25 or 0.25.

With respect to data on costs of reductions in nutrient loads and removal by mussel farms, data on the cost of nutrient load reductions in each country are found in Gren and Elofsson [3], who constructed a cost minimisation model for reductions in nutrient loads to the Baltic Sea. The cost minimisation model included costs and effects from different abatement measures in the agricultural sector, wastewater treatment and airborne emissions. Measures in the agricultural sector included increases in grassland and energy crops, cultivation of catch crops, reductions in the use of fertilisers and livestock holdings of cattle, pigs and chicken, the increase in the storage of manure, and the creation of wetlands and buffer strips. Sewage treatment is improved through cleaning at sewage treatment plants, industrial and private sewers, and phosphorus-free detergents, and airborne emissions are reduced by increased reduction in nitrogen oxides in transport, energy production and industry.

The costs of nutrient abatement by mussel farming were determined by i) mussel growth, ii) the cost of labour, capital and equipment used for establishing and managing a farm, iii) technical life length of the farm and discount rate, and iv) income from the sale of mussels as food, feed, inputs as fertiliser, or as an energy source. These four factors determine the net cost of mussels for nutrient removal, which differ between countries and regions. The cost is also likely to be positive and increasing at each site, i.e. the unit cost of mussel production increases as more farms enter a specific site because of lower productivity. This is not examined in any of the existing studies on costs of mussel farming which assume a linear relation where marginal and average cost are constant. Except for Gren *et al*. [5], existing studies calculate costs for farms at the local scale, such as the cultivation of zebra mussels (*Dreissena polymorpha*) in the Oder lagoon in Germany [6] or blue mussels (*Mytilus edulis*) on the west coast of Sweden [4]. The present study used the estimates made by [5], who performed a systematic calculation of costs of mussel farming in the Baltic Sea region for blue mussels with the commonly used long line technology. Labour costs were adjusted to the average salary in the agricultural sector in each country. Common assumptions for all countries were a technical life length of 10 years and a discount rate of 5%. One difference with the cost estimates in Gren et al. [5] is the exclusion of incomes of sales of mussels, because of the uncertainty with respect to availability of markets for food, feed and energy. The costs were adjusted to the 2015 price level in each country.

The calculated range in annual costs of a mussel farm at the 10 different sites was between 0.010 and 0.032 million Euro per annum and farm of size 0.5 ha, being lowest in Russia and highest in Denmark (S2 Table). The costs per kg live mussels varied between 0.12 Euro/kg (Russia) and 0.41 Euro/kg (Sweden, North Baltic Proper). The estimate for Denmark, which amounted to 0.18 Euro/kg, can be compared with similar calculations made by Ngyuen *et al*. [12] and Petersen *et al*. [13], who found that the cost of cultivating blue mussels in the Skive fjord amounted to 0.14 Euro/kg and in the Great Belt to 0.24 Euro/kg (at 2015 prices). The calculated unit cost for Sweden in the Kattegat Basin, which amounted to 0.17 Euro/kg, can be compared with estimates made by Lindahl *et al*. [4], who calculated a cost of 0.10 Euro/kg for blue mussel farming on the west coast of Sweden (at 2015 prices).

Finally, there was a need to determine the targets to be achieved, and the choice of probabilities of achieving these targets. The international agreement in the Baltic Sea Action Plan (BSAP) on nutrient loads suggests a reduction of 42% in the load of phosphorus and 14% in nitrogen loads to the Baltic Sea [21]. Due to this large reduction in phosphorus, the capacity constraints on abatement measures, and the high risk discount with the Chebyshev’s inequality a relatively modest probability, *α = 0*.*6*, of achieving both nutrient targets was assigned. This gives a *ϕ*^*α*,*U*^ = 0.23 for a normal distribution and *ϕ*^*α*,*U*^ = 1.58 with Chebyshev’s inequality. It was then possible to compare outcomes under different assumptions of probability distribution and covariation between nutrient abatement by mussel farming and other abatement measures.

## Results: Calculated value of mussel farming

The numerical cost minimisation problem is solved using GAMS with the CONOPT solver [33]. Without uncertainty and mussel farming, the total minimum cost for achieving the BSAP targets amounts to 3.44 billion Euro, which corresponds to approximately 0.3% of total GDP in the catchment in 2015 [34]. The marginal costs of other abatement measures without any mussel farming at the nutrient targets, the so-called shadow costs of the targets, amount to 9.51 Euro/kg N reduction and 412.07 Euro/kg P reduction. The first test of whether or not nutrient removal by mussel farming has a value is if the marginal removal cost is below these marginal abatement costs (Table 2).

**Table 2:** Shadow costs^a^ of reaching nutrient targets without mussel farming and marginal nutrient removal cost of mussel farming, Euro/kg nitrogen (N) and phosphorus (P).

	No uncertainty: N P		Uncertainty: Normal distribution Chebyshev’s ineq. N P N P			
Shadow cost of nutrient targets	9.51	412.07	10.68	469.01	21.47	1536.33
Marginal removal cost of mussel farming	12.50–41.43	156.25–517.86	13.45–44.23	166.78–552.77	17.57–58.25	219.45–727.33

^a^Increase in total minimum abatement cost of increasing abatement target by 1 kg

The marginal costs of nutrient removal by mussel farming were calculated for each country and catchment based on the data on production and costs of mussels in Table 1 and S2 Table. The marginal risk discount corresponded to the standard at the probability of 0.6, which amounted to 0.26 for the normal distribution and to 1.58 for Chebyshev’s inequality, times the coefficient of variation in mussel production displayed in Table 1. The increases in marginal removal costs under uncertainty then ranged between 12% and 21%, depending on the coefficient of variation.

The results in Table 2 show that the marginal costs of nitrogen removal by mussel farming are below the marginal costs of other abatement measures only for Chebychev’s inequality. On the contrary, the lower range of the marginal cost of phosphorus removal by mussel farming is always below that of other abatement measures. The reason for this difference in the economic performance of mussel farming is the differences in the stringency of the nutrient reduction targets, which is 14% for nitrogen and 42% for phosphorus, and the relatively high costs of reductions in phosphorus loads. This is also shown by the differences on abatement costs with and without mussel farming for separate targets on nitrogen and phosphorus, where the costs of phosphorus reductions are five to six times higher than for nitrogen (S3 Table).

Below is a calculation of the value of mussel farming, as shown in Section 2, in three different scenarios:

- scenario 1: no uncertainty, which is common in practice (*e*.*g*. HELCOM [21])
- scenario 2: uncertainty only in nutrient removal by mussel farming, which is discussed as a potential drawback of the measure (*e*.*g*. Stadmark and Conley [7])
- scenario 3: uncertainty in all abatement measures, as demonstrated in Section 2.

**Fig 1:**
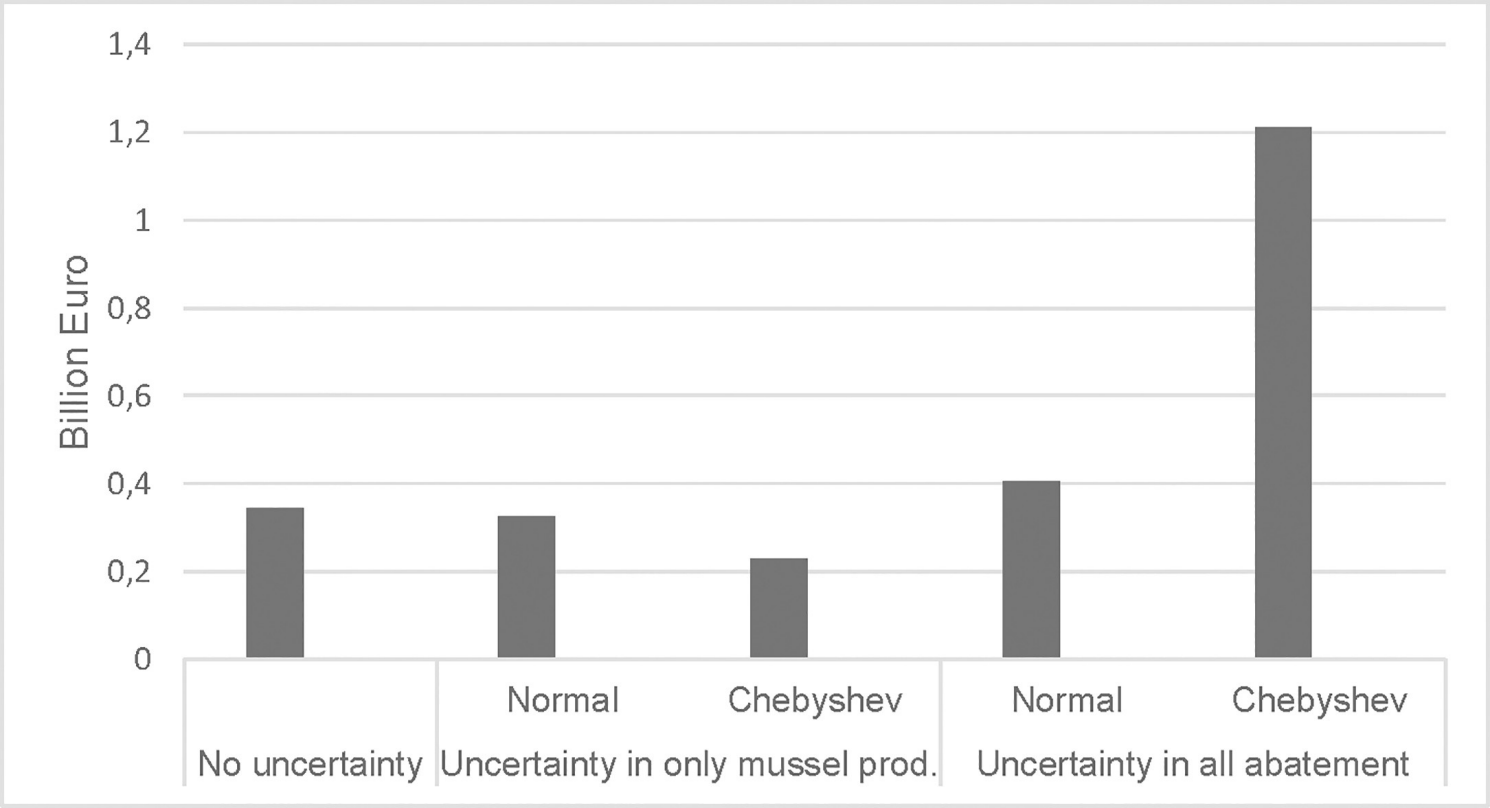
Value of mussel farming of reaching BSAP nutrient reduction targets under different combinations of uncertainty and assumptions of probability distributions (normal and Chebyshev’s inequality). Source: S4 Table.

The value of mussel farming, calculated as the minimum costs of reaching the nutrient targets with and without mussel farming, under these three scenarios are presented (Fig 1).

Without uncertainty, the introduction of mussel farming as an abatement measure reduces the overall cost of reaching the targets by 0.34 billion Euro, or 10% of total cost. The value of mussel farming is reduced to 0.23 billion Euro when uncertainty is attributed only to this measure and Chebyshev’s inequality is assumed for the probability distribution. This probability distribution raises the cost considerably with and without mussel farming because of the high risk discount. The value of mussel farming in terms of cost savings can then correspond to 1.21 billion Euro. The relatively high risk discount for a probability of 0.6 under Chebyshev’s inequality corresponds to a probability of 0.94 when a normal probability distribution is assumed. The higher cost of reaching the targets and associated value of mussel farming can then be interpreted as impacts under a normal probability distribution when raising the probability of achieving the nutrient targets from 0.6 to 0.94.

The results presented in Fig 1 are based on the assumption of a zero covariation between nutrient removal by mussel farming and abatement by other measures. The existence of a positive or negative correlation (a correlation coefficient of 0.25 or -0.25, respectively) has a relatively small impact on the value of mussel farming with a normal distribution. The negative covariation can increase the value by 83% with Chebyshev’s inequality (S4 Table) because of the hedging against low impacts of abatement in the catchment. On the other hand, when occasions with low abatement by mussel farming and in the catchments coincide the value approaches zero since its inclusion increases the variance in total abatement.

However, although the total value of mussel farming for all countries is positive, not all countries gain from its introduction (Table 3).

**Table 3:** Calculated value of mussel farming for nutrient removal in the Baltic Sea for different countries under different uncertainty scenarios (billion Euro).

Countries	No uncertainty	Uncertainty only in mussel production: Normal Chebyshev		Uncertainty in all abatement: Normal Chebyshev	
DEN	-0.091	-0.091	-0.094	-0.089	-0.022
EST	0.016	0.015	0.010	0.020	0.003
FIN	0.014	0.014	0.012	0.015	0.193
GER	-0.078	-0.078	-0.079	-0.078	-0.029
LAT	0.041	0.040	0.032	0.047	0.096
LIT	0.072	0.070	0.058	0.083	0.035
POL	0.313	0.303	0.252	0.348	0.721
RUS	0.080	0.078	0.065	0.082	0.092
SWE	-0.025	-0.026	-0.029	-0.023	0.124
Total	0.343	0.324	0.226	0.405	1.212

The impact of mussel farming on a country is explained by two factors; the overall abatement effect and the allocation effect. All countries gain from the overall abatement effect where mussel farming replaces more expensive abatement measures. The allocation effect implies that abatement by mussel farming takes place in countries with the lowest cost and large mussel farming capacities. The reason for the large value to Poland is the abatement size effect in terms of a decrease in the country’s abatement owing to the phosphorus removal carried out by mussel farming. This, in turn, is explained by Poland’s large share of nutrient loads and the relatively low abatement cost of other measures. When mussel farming is introduced, the allocation effect implies that mussel farming is placed mainly in Denmark, Germany, and Sweden, which explains the higher abatement costs and hence negative value of mussel farming in these countries.

## Discussion

As in all quantitative analyses, the results depend on the assigned parameter values and chosen model construction. Nevertheless, the calculated total minimum cost for reaching the BSAP nutrient targets without mussel farming of 3.44 billion Euro is in the same order of magnitude as the estimates obtained for reaching the same BSAP targets, but with other numerical optimisation models [35, 36]. The costs obtained by Elofsson [35] and Hasler *et al*. [36] amount to 3.74 and 4.06 billion Euro, respectively. The lower cost in the current study is explained by the inclusion of a larger number of abatement measures, such as the reduction in airborne emissions, and the consideration of overall nutrient load targets rather than specific targets for each marine basin.

**Fig 2:**
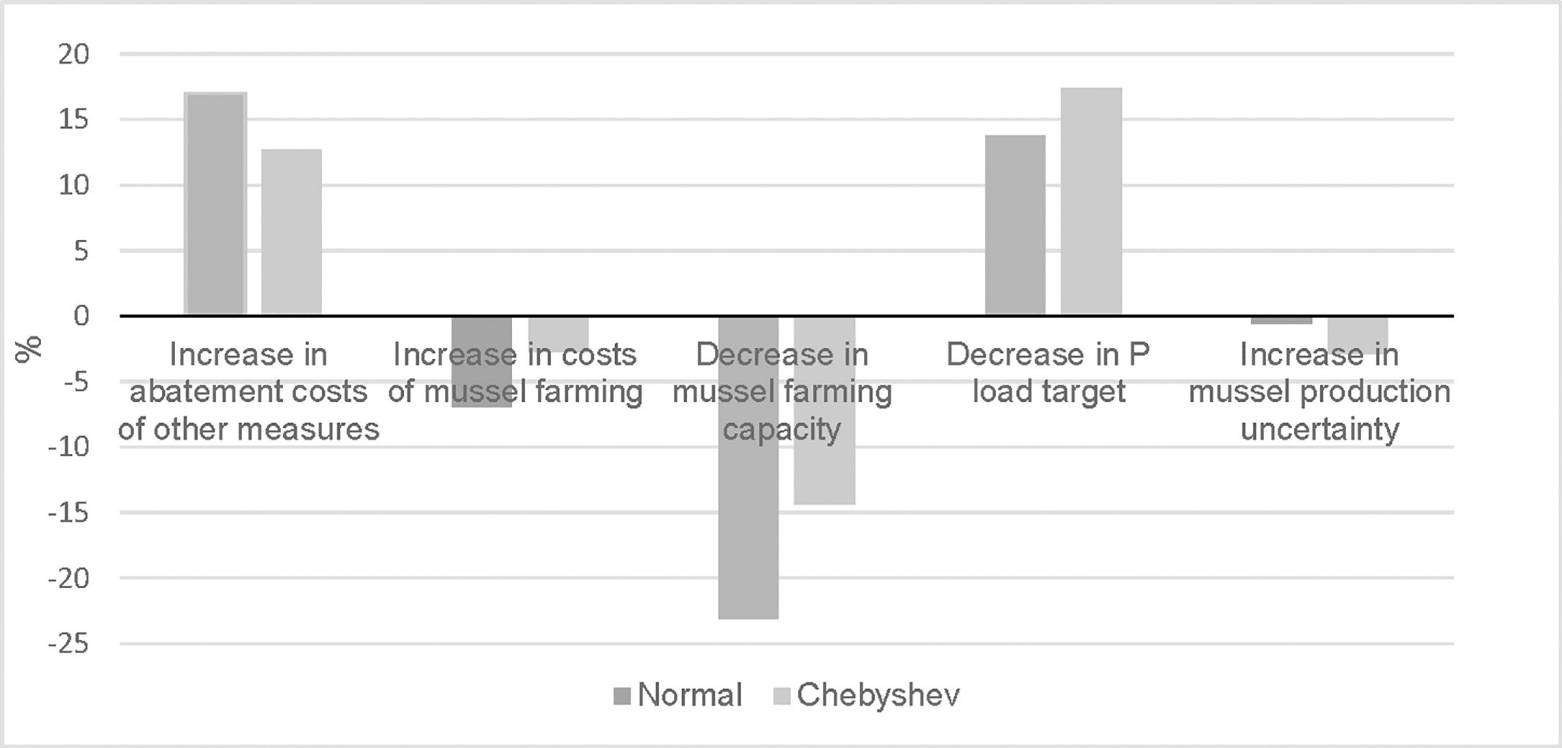
Sensitivity in the value of mussel farming of different parameters, measured as % change in value from a 10% change in each parameter when all nutrient abatement is uncertain under different probability distributions (normal and Chebyshev). Source: S5 Table.

The value of mussel farming increases when the costs of other abatement measures increase, the cost of mussel farming decreases, the mussel production capacity increases, and nutrient targets are made more stringent. In order to calculate the sensitivity in the value of mussel farming to changes in these parameters, elasticities were calculated, which showed the percentage change in the value from a 10% change in these parameters (Fig 2).

The results displayed in Fig 2 show that the elasticity is relatively low for both probability distribution when costs of mussel farming and uncertainty in abatement by mussel farming change. Instead, higher costs of other abatement measures and a reduction in the maximum load of phosphorus raise the value by at least 13%. On the other hand, when the available capacity of mussel farming decreases by 10%, the value decreases by up to 23% since less nutrient abatement by other measures can be replaced. The available capacity can decrease because of lower mussel growth and/or less area available for mussel farming.

The numerical results depend not only on parameter values in the constructed optimisation model, but on underlying model limitations and assumptions as well. One is the static model, which does not consider any dynamics and future impacts in the biological/hydrological or economic systems, such as assimilation of nutrients in the sea or transports in the catchments, and technological and economic development. Another is the disregard of other environmental impacts of mussel farming and other abatement measures, such as climate and biodiversity impacts. An important assumption is that the costs of nutrient reductions are minimised for all countries. The existing international agreement envisages separate country targets, which are likely not to coincide with the cost-effective solutions (*e*.*g*. [35]). Whether or not these model limitations and assumptions affect the value of mussel farming and its allocation between the countries is an empirical question.

## Conclusions

The main conclusion drawn from this study is that the consideration of uncertainty in all nutrient abatement increases the value of nutrient removal by mussel farming in the Baltic Sea from 0.34 billion Euro per year to 0.41 or 1.21 billion Euro depending on assumption of probability distribution. There are two main reasons for this increase. One is that the consideration of uncertainty is expressed in a safety-first setting, which implies that the target becomes more stringent because of the risk discounting of uncertain abatement. This implies that the cost of reaching nutrient targets without mussel farming becomes relatively high. The other reason is that the uncertainty as measured by coefficient of variation is slightly lower for nutrient removal by mussel farming than for other abatement measures, which implies a relative cost advantage for this abatement measure.

The results also showed that the calculated value is sensitive to, in particular, measurement of uncertainty and level of risk aversion. The value of mussel farming can increase by 200% if the probability distribution is expressed as a Chebyshev’s inequality instead of a normal distribution. Similarly, the value increases by the same magnitude when the risk aversion increases as expressed as a higher probability of reaching the target when a normal probability distribution is assumed. These results support findings from similar studies (*e*.*g*. [9,10,11]) on the need to define and measure risk aversion and uncertainty in reaching environmental targets.

The estimated value was also sensitive to changes in mussel production capacity. The future value of mussel farming as a nutrient abatement measure can then decrease since climate change is expected to decrease the salinity levels in the Baltic Sea [37]. Salinity levels close to 6 practical salinity unit (psu) are regarded as particular harmful to mussel growth [26]. Mussel yield is then reduced and may approach zero at farms in the coastal zones of the Baltic Proper where average salinity levels range between 6 and 8 psu [38]. On the other hand, climate is also expected to increase nutrient loads from the catchment (*e*.*g*. [39]) which might promote mussel growth because of increased food availability [40]. The results in this study showed that the value of mussel farming can increase considerably when nutrient removal by mussel farming is high when nutrient loads from the catchment are large. Mussel farming than acts as a hedge against large nutrient runoff from the catchment. This points to the need to consider uncertainty in all abatements, and not only in the new cleaning technology, mussel farming in this study.

However, the total value was unevenly distributed between the countries due to differences in salinity levels, the length of coastal zones, and costs of labour and capital. High salinity levels and long coastlines are found in Denmark, Germany and Sweden, which implies that these countries make losses from the introduction of mussel farming because the cost of implementing mussel farms are higher than the gains from cost savings by reductions from other abatement measures. However, all the other countries, in particular Poland, make gains from cost savings. As shown by Gren *et al*. [15] this effect implies that mussel farming promotes fairness in the allocation of abatement cost burdens among the countries.

Although mussel farming provides a value to society in terms of less expensive cleaning of the Baltic Sea, the implementation of this in practice requires payments to mussel farmers that, at least, cover the costs of farming. One option is to give support to mussel farms similar to the EU’s CAP (Common Agricultural Policy) payment for wetland construction. Another is to allow firms subject to nutrient regulations schemes, such as the BSAP and Water Framework Directive, to use mussel farming as an offset, paying mussel farmers to carry out some of their reduction requirements. Admittedly, this analysis did not consider costs associated with monitoring and verifying nutrient removal, which are necessary for exchanges in nutrient abatement between mussel farmers and actors with other abatement measures. The inclusion of such so-called transaction costs will increase the costs of mussel farming, which needs to be considered and calculated in order to verify whether mussel farming still remains a profitable nutrient abatement option for society.

## Supporting information

S1 Appendix Derivation of conditions for nutrient abatement value of mussel farming. (DOCX)

S1 Table Mussel production in ton/ha in different studies and locations. (DOCX)

S2 Table Annual cost of mussel farm^a^, straight coastlines in km, potential mussel production area in ha^b^ and mussel production in ktonne/per year^c^. (DOCX)

S3 Table Minimum costs for separate emission targets on nitrogen, N, and phosphorus, P, billion Euro. (DOCX)

S4 Table Minimum costs of nutrient abatement targets with and without mussel farming and different combinations of uncertainty. (DOCX)

S5 Table Minimum nutrient abatement costs for reaching BSAP nutrient targets with and without mussel farming with 10% changes in different parameters for normal and Chebyshev probability when all abatement is uncertain. (DOCX)

S1 Fig The Baltic Sea drainage and marine basin. Source: GRID Arendal (http://www.grida.no/baltic/htmls/maps.htm).
(TIF)
