# Supplementary material for: The economic value of mussel farming for uncertain nutrient removal in the Baltic Sea
Source: PLoS One. 2019 Jun 14;14(6):e0218023. doi: 10.1371/journal.pone.0218023 (PMC6570029; doi:10.1371/journal.pone.0218023)
Supplement: S1 Appendix — (DOCX) [file pone.0218023.s001.docx]

**S1 Appendix. Derivation of conditions for nutrient abatement value of**

**mussel farming**

The first-order conditions for a cost effective solution to the decision problem defined by eq. (8) are obtained by constructing the Lagrange expression, which gives:

$L=\sum_{i} C^{i,M}+C^{i,O}+\sum_{U} \lambda^{U}(E\left[A^{U}\right]-\varphi^{\alpha,U}(\sigma^{U})^{1/2}-\overline{A}^{U})+\beta^{i,M}(\overline{A}^{i,M}-A^{i,M})+\beta^{i,O}(\overline{A}^{i,O}-A^{i,O})$ (A1)

where $E\left[A^{U}\right]=\sum_{i} c^{i,U,M}A^{i,M}+c^{i,U,O}A^{i,O}$, *σ^U^=Var(A^U^)*, $\lambda^{U}\geq0$ are the Lagrange multipliers on the nutrient targets, and $\beta^{i,M}\leq0$and $\beta^{i,O}\leq0$are the Lagrange multipliers on the capacity constraints on nutrient removal by mussel farms and abatement by other measures, respectively. The first-order conditions are obtained by differentiating eq. (A1) with respect to *A^i,M^* and *A^i,O^* which gives:

$\frac{\partial C^{i,M}}{\partial A^{i,M}}=\sum_{U} \lambda^{U}\left(-c^{i,U,M}-\frac{\varphi^{\alpha,U}}{2(\sigma^{U})^{1/2}}\frac{\partial\sigma^{U}}{\partial A^{i,M}}\right)+\beta^{i,M}$ *for i=1,…,g*  (A2)

$\frac{\partial C^{i,O}}{\partial A^{i,,O}}=\sum_{U} \lambda^{U}\left(-c^{i,U,O}-\frac{\varphi^{\alpha,U}}{2(\sigma^{U})^{1/2}}\frac{\partial\sigma^{U}}{\partial A^{i,O}}\right)+\beta^{i,O}$ *for i=1,..,g*  (A3)

The first-order condition for each type of measure shows that marginal abatement costs of the measure, the term on the left-hand side of (A2) and (A3), should equal the marginal impact on the target weighted by the Lagrange multipliers *λ^U^* . For analytical convenience, but without loss of generality, interior solutions were assumed where the capacity constraints on mussel farming and nutrient abatement by other measures are non-binding. A cost-effective solution then requires that marginal nutrient reduction costs are equal for the two options, which is shown for nitrogen *N* as:

$\left(\frac{\partial C^{i,M}}{\partial A^{i,M}}-MP^{i,M}\right)/\left(c^{i,N,M}-MR^{i,N,M}\right)=\lambda^{N}=\left(\frac{\partial C^{i,O}}{\partial A^{i,O}}-MP^{i,O}\right)/\left(c^{i,N,O}-MR^{i,N,O}\right)$ (A4)

where $MP^{i,P,M}=\lambda^{P}\left(c^{i,P,M}-MR^{i,P,M}\right)$ and$MP^{i,P,O}=\lambda^{P}\left(c^{i,P,O}-MR^{i,P,O}\right)$ are the marginal impacts on phosphorus abatement, $MR^{i,N,M}=\frac{\varphi^{\alpha,N}}{2(\sigma^{N})^{1/2}}\frac{\partial\sigma^{N}}{\partial A^{i,M}}$ and $MR^{i,N,O}=\frac{\varphi^{\alpha,N}}{2(\sigma^{N})^{1/2}}\frac{\partial\sigma^{N}}{\partial A^{i,O}}$ are the marginal impacts on the risk discount of mussel farming and other abatement measures respectively. Mussel farming then has a value in terms of cost savings only if:

$\left(\frac{\partial C^{i,M}}{\partial A^{i,M}}-MP^{i,M}\right)/\left(c^{i,N,M}-MR^{i,N,M}\right)<\left(\frac{\partial C^{i,O}}{\partial A^{i,O}}-MP^{i,O}\right)/\left(c^{i,N,O}-MR^{i,N,O}\right)$ (A5)

evaluated at *A^i,M^=0.*
