# Supplementary material for: The economic value of mussel farming for uncertain nutrient removal in the Baltic Sea
Source: PLoS One. 2019 Jun 14;14(6):e0218023. doi: 10.1371/journal.pone.0218023 (PMC6570029; doi:10.1371/journal.pone.0218023)
Supplement: S1 Table — (DOCX) [file pone.0218023.s002.docx]

**S1 Table. Mussel production in ton/ha in different studies and locations**

| **Study** | **East coast of Denmark/ west coast of Sweden** | **Baltic Proper** |
| --- | --- | --- |
| Lindahl *et al*. [4] | 462-594 |  |
| Gren *et al.* [5] | 180-350 | 140-160 |
| Ngyen *et al*. [12] | 140 |  |
| Petersen *et al*. [13 ] | 60-90 |  |
| Schernewski *et al*. [14] |  | 83-167 |
| Haamer [28] | 200 |  |
| Lindahl and Kollberg [29] | 200-300 | 40-90 |
