# Supplementary material for: The economic value of mussel farming for uncertain nutrient removal in the Baltic Sea
Source: PLoS One. 2019 Jun 14;14(6):e0218023. doi: 10.1371/journal.pone.0218023 (PMC6570029; doi:10.1371/journal.pone.0218023)
Supplement: S2 Table — (DOCX) [file pone.0218023.s003.docx]

**S2 Table. Annual cost of mussel farm^a^, straight coastlines in km, potential mussel production area in ha^b^ and mussel production in ktonne/per year^c^**

| **Countries** | **Million Euro/ farm^g^** | **North Baltic Proper:**  **km ha ktonne** | | | **South Baltic Proper:**  **km ha ktonne** | | | **Kattegat and the Danish Straits:**  **km ha ktonne** | | |
| --- | --- | --- | --- | --- | --- | --- | --- | --- | --- | --- |
| DEN^d^ | 0.032 |  |  |  |  |  |  | 1701 | 1573 | 551 |
| EST^d^ | 0.014 | 621 | 575 | 80 |  |  |  |  |  |  |
| GER^e^ | 0.026 |  |  |  | 669 | 618 | 99 | 1341 | 1240 | 434 |
| LAT^d^ | 0.012 |  |  |  | 498 | 461 | 74 |  |  |  |
| LIT^d^ | 0.012 |  |  |  | 90 | 83 | 13 |  |  |  |
| POL^e^ | 0.013 |  |  |  | 634 | 586 | 94 |  |  |  |
| RUS^f^ (Kalin-ingrad) | 0.010 |  |  |  | 143 | 132 | 21 |  |  |  |
| SWE^d^ | 0.029 | 821 | 759 | 106 |  |  |  | 788 | 728 | 255 |
| Total |  | 1442 | 1334 | 186 | 1881 | 2015 | 301 | 3830 | 3542 | 1240 |

^a^ Assumption of technical life length of 10 years and a discount rate of 5%; ^b^Calculated by an assumed maximum mussel area corresponding to 0.5 % of the area within 1 nm (1.85 km) of the coastline; ^c^ average mussel production per farm of size 0.5 ha, which is 175 tonnes in Kattegat/Danish Straits, 70 tonnes in North Baltic Proper, and 80 tonnes in South Baltic Proper [5]; ^d^coastline data from CIA [41]; ^e^coastline data from EC [42]; ^f^coastline data from CCB [43]; ^g^Gren *et al.* [5] farm of size 0.5 ha,

**References**

[41] CIA (Central Intelligence Agency) 2018. The World Fact Book. [cited 28 October 2018]

Available from <https://www.cia.gov/library/publications/the-world-factbook/fields/2060.html>

[42] EC (European Commission) 2018. Country reports and assessments. [cited 28 October 2018] . Available from <https://ec.europa.eu/maritimeaffairs/sites/maritimeaffairs/files/docs/body/germany_climate_change_en.pdf>

[43] CCB (Coalition Clean Baltic) 2011. National report concerning the Baltic green belt (the coastal zone), Russia). [cited 28 October 2018]. Available from

<https://www.ccb.se/documents/Nationalreport_RUSSIA.pdf>
