# Supplementary material for: The economic value of mussel farming for uncertain nutrient removal in the Baltic Sea
Source: PLoS One. 2019 Jun 14;14(6):e0218023. doi: 10.1371/journal.pone.0218023 (PMC6570029; doi:10.1371/journal.pone.0218023)
Supplement: S3 Table — (DOCX) [file pone.0218023.s004.docx]

**S3 Table. Minimum costs for separate emission targets on nitrogen, N, and**

**phosphorus, P, billion Euro**

|  | **No uncertainty:**  **N P** | | **Uncertainty, normal:**  **N P** | | **Uncertainty, Chebyshev:**  **N P** | |
| --- | --- | --- | --- | --- | --- | --- |
| No mussel | 0.60 | 3.18 | 0.67 | 3.61 | 1.32 | 8.45 |
| With mussel | 0.60 | 2.91 | 0.67 | 3.29 | 1.30 | 7.38 |
