# Supplementary material for: The economic value of mussel farming for uncertain nutrient removal in the Baltic Sea
Source: PLoS One. 2019 Jun 14;14(6):e0218023. doi: 10.1371/journal.pone.0218023 (PMC6570029; doi:10.1371/journal.pone.0218023)
Supplement: S4 Table — (DOCX) [file pone.0218023.s005.docx]

**S4 Table. Minimum costs of nutrient abatement targets with and without mussel farming and different combinations of uncertainty**

|  | **No uncertainty** | **Uncertainty in only mussel prod.:**  **Normal Chebyshev** | | **Uncertainty in all abatement:**  **Normal Chebyshev** | |
| --- | --- | --- | --- | --- | --- |
| No mussel farming | 3.44 | 3.44 | 3.44 | 3.90 | 8.98 |
| Mussel farming | 3.09 | 3.11 | 3.21 | 3.49 | 7.77 |
| Correlation coefficient = -0.25 | | | | 3.42 | 6.77 |
| Correlation coefficient = 0.25 | | | | 3.56 | 8.87 |
