# Supplementary material for: The economic value of mussel farming for uncertain nutrient removal in the Baltic Sea
Source: PLoS One. 2019 Jun 14;14(6):e0218023. doi: 10.1371/journal.pone.0218023 (PMC6570029; doi:10.1371/journal.pone.0218023)
Supplement: S5 Table — (DOCX) [file pone.0218023.s006.docx]

**S5 Table. Minimum nutrient abatement costs for reaching BSAP nutrient targets with and without mussel farming with 10% changes in different parameters for normal and Chebyshev probability when all abatement is uncertain.**

|  | **Reference value** | **Increase in abatement costs of other measures** | **Increase in costs of mussel farming** | **Decrease in mussel farming capacity** | **Reduction in P abatement target** | **Uncertainty increase in mussel farming** |
| --- | --- | --- | --- | --- | --- | --- |
| Normal: |  |  |  |  |  |  |
| No mussel farming | 3.896 | 4.286 | 3.896 | 3.896 | 3.270 | 3.896 |
| Mussel farming | 3.491 | 3.812 | 3.519 | 3.585 | 2.920 | 3.494 |
| Chebyshev: |  |  |  |  |  |  |
| No mussel farming | 8.978 | 9.876 | 8.978 | 8.978 | 7.339 | 8.978 |
| Mussel farming | 7.765 | 8.509 | 7.798 | 7.939 | 6.337 | 7.801 |
