# Supplementary figures and images for: The economic value of mussel farming for uncertain nutrient removal in the Baltic Sea
Source: PLoS One. 2019 Jun 14;14(6):e0218023. doi: 10.1371/journal.pone.0218023 (PMC6570029; doi:10.1371/journal.pone.0218023)

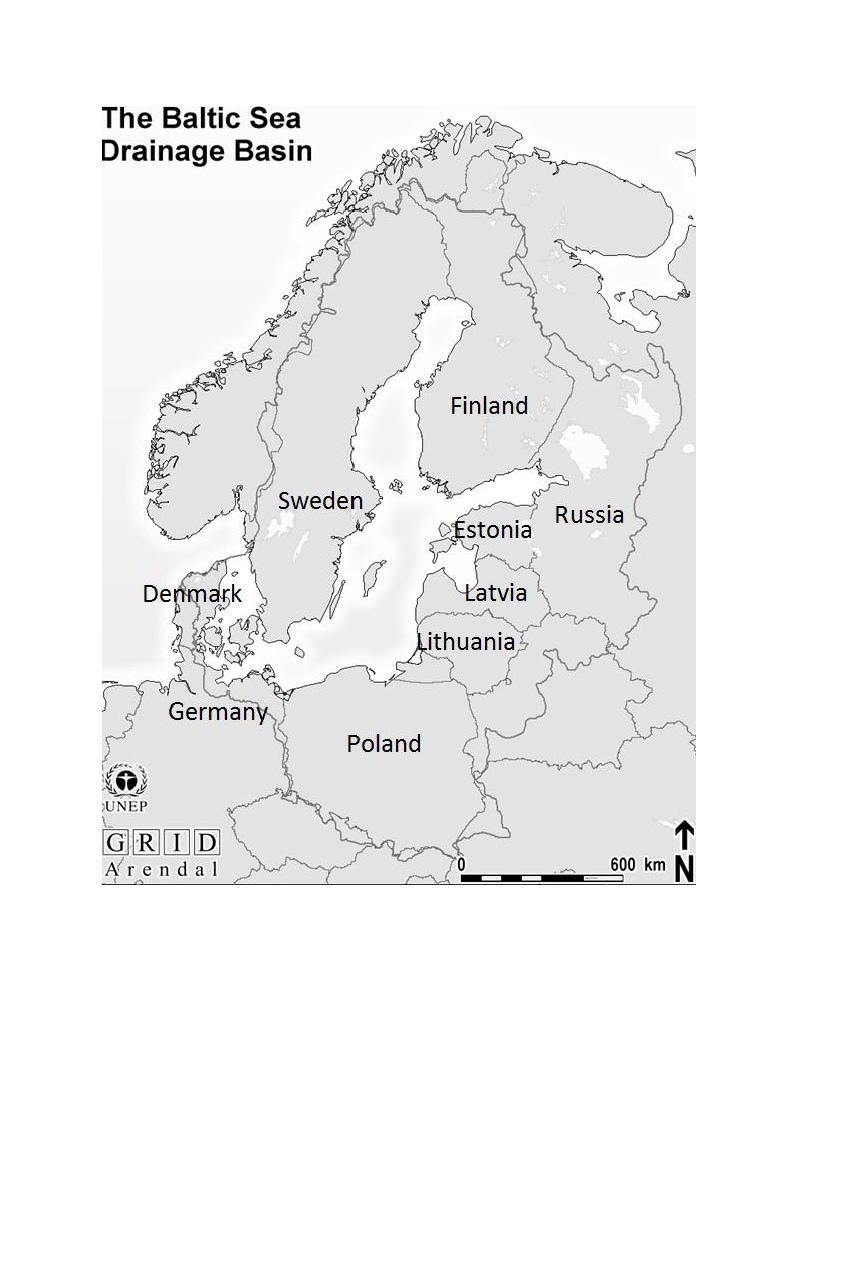

Supplement: S1 Fig — Source: GRID Arendal (http://www.grida.no/baltic/htmls/maps.htm). (TIF) [file pone.0218023.s007.tif]
